# An evolving roadmap: using mitochondrial physiology to help guide conservation efforts

**DOI:** 10.1093/conphys/coae063

**Published:** 2024-09-07

**Authors:** Elisa Thoral, Neal J Dawson, Stefano Bettinazzi, Enrique Rodríguez

**Affiliations:** Department of Biology, Section for Evolutionary Ecology, Lund University, Sölvegatan 37, Lund 223 62, Sweden; School of Biodiversity, One Health and Veterinary Medicine, University of Glasgow, Garscube Campus, Bearsden Road, Glasgow, G61 1QH , UK; Research Department of Genetics, Evolution and Environment, University College London, Darwin Building, 99-105 Gower Street, WC1E 6BT, London, UK; Research Department of Genetics, Evolution and Environment, University College London, Darwin Building, 99-105 Gower Street, WC1E 6BT, London, UK

**Keywords:** Bioenergetics, climate change, conservation, ecophysiology, mitochondria

## Abstract

The crucial role of aerobic energy production in sustaining eukaryotic life positions mitochondrial processes as key determinants of an animal's ability to withstand unpredictable environments. The advent of new techniques facilitating the measurement of mitochondrial function offers an increasingly promising tool for conservation approaches. Herein, we synthesize the current knowledge on the links between mitochondrial bioenergetics, ecophysiology and local adaptation, expanding them to the wider conservation physiology field. We discuss recent findings linking cellular bioenergetics to whole-animal fitness, in the current context of climate change. We summarize topics, questions, methods, pitfalls and caveats to help provide a comprehensive roadmap for studying mitochondria from a conservation perspective. Our overall aim is to help guide conservation in natural populations, outlining the methods and techniques that could be most useful to assess mitochondrial function in the field.

## Teaser

Mitochondrial processes play crucial roles in species’ responses to environmental variation. Here, we synthesize the methods and approaches involved in mitochondrial bioenergetics as tools to guide the field of conservation physiology.

## Graphical Abstract

**Graphical Abstract:**
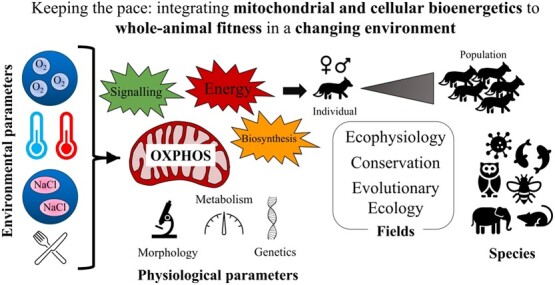

## Introduction

The study of metabolism integrates fundamental physicochemical principles and biology, to connect organismal physiology to the ecology of populations, environments and ecosystems. Metabolic rate, the rate at which organisms use energy, is considered a fundamental organismal trait: considered the ‘cost of living’ that unifies all levels of biological organization (Brown *et al.*, 2024). For decades, investigations of the causes and consequences of variation in metabolic rate, both among and within species using whole-animal oxygen consumption rates have dominated the field of conservation biology and ecology (Biro and Stamps, 2010; Burton *et al.*, 2011; Glazier, 2015; Pettersen *et al.*, 2018). However, whole-organismal measures of metabolic rates have limitations for a number of reasons, including: (i) an unknown proportion of the consumed oxygen is actually associated with energy production in the form of ATP; (ii) the inability to determine where variation in oxygen consumption takes place and (iii) typically involve measuring maximum aerobic capacity, where oxygen supply may be a limiting factor. As a result, measures of total oxygen consumption frequently fail to find clear links to behaviours like wild fish activity (Baktoft *et al.*, 2016), exploratory behaviour in wild mammals (Timonin *et al.*, 2011), risk-taking in wild birds (Mathot and Dingemanse, 2015), temperature tolerance in ectotherms (Jutfelt *et al.*, 2018), reproductive output in mice (Duarte *et al.*, 2010) or rates of ageing in wild birds (Bouwhuis *et al.*, 2011). Natural selection might act on energetic efficiency, rather than oxygen consumption; where individuals expending minimal energy to sustain these traits are likely to be favoured over individuals with high rates of energy or oxygen consumption. To address this issue, it is crucial to investigate the functioning of mitochondria within animals in their natural habitats.

For almost a century, the study of mitochondrial energy production has been an essential tool in physiology, as evidenced by more than a quarter million publications since 1925 including the keywords ‘mitochondria’ and ‘physiology’ (Web of Science). With the advent of new techniques allowing a more in-depth study of mitochondrial phenotype (Gnaiger and MitoEAGLE Task Group, 2020), biologists from different fields are increasingly interested in mitochondrial bioenergetics. Indeed, the measurement of mitochondrial function has extended across a wide range of disciplines, including ecophysiology, evolutionary ecology and conservation biology, as ways to gain insights into the mechanisms underpinning variation in life-history phenotypes (Koch *et al.*, 2021). Conservation physiology uses physiological theory and tools to study how environmental perturbations link to ecological performance of vulnerable species and populations (Seebacher and Franklin, 2012). Among the physiological parameters of interest, mitochondrial metabolism appears to, at least partly, dictate the capacity of animals to face changes in environmental conditions (Iftikar *et al.*, 2014; Jørgensen *et al.*, 2021). Therefore, predicting the extent of mitochondrial and energy metabolism plasticity in response to the environment should contribute to the successful identification of vulnerable species and dictate the necessary interventions for conservation.

While it is out of the scope of this review to provide a detailed explanation of mitochondrial function, researchers investigating the role of mitochondrial bioenergetics in a conservation context can refer to past reviews on the precise mechanisms behind this organelle’s function (Wallace and Fan, 2010; Gnaiger and MitoEAGLE Task Group, 2020). However, as interest in harnessing mitochondrial physiology as a means to guide conservation efforts continues to develop, careful consideration should be given to adapting protocols designed in model organisms (Rodríguez *et al.*, 2023) for use in the field (Nord *et al.*, 2021; Parry *et al.*, 2021). It is also critical to understand how and when to use the many different parameters underpinning mitochondrial phenotypes (e.g. oxygen flux, ATP production, reactive oxygen species, membrane potential, cristae morphology) to properly determine how cellular energy production links organismal performance to the ecology of populations, environments and ecosystems (Metcalfe *et al.*, 2023). Consequently, it is essential to match measured mitochondrial parameters to the specific conservation outcomes and question(s) asked, as well as which species and tools are most appropriate for a given ecological niche.

## Integrating mitochondrial and cellular bioenergetics to whole-animal fitness in a changing environment

A wide variety of approaches are used to study mitochondrial function, as can be appreciated by the different biological preparations involved, from isolated mitochondria (Rhodes *et al.*, 2024), to intact cells (Nord *et al.*, 2023), permeabilized tissue (Dawson *et al.*, 2016) or shredded tissue samples (Thoral *et al.*, 2021). The parameters measured can also vary, ranging from structure and morphology (Bock *et al.*, 2019; Rodríguez *et al.*, 2019; Christen *et al.*, 2020), to oxygen consumption (Teulier *et al.*, 2019), ATP production (Barbe *et al.*, 2023b), membrane potential (Harford *et al.*, 2023) as well as by-products of metabolism, such as reactive oxygen species (ROSs) production (Christen *et al.*, 2018). In addition to energy production, mitochondrial function is essential in the maintenance of homeostasis, with fusion/fission dynamic, calcium regulation, heat production in endotherms (Nord *et al.*, 2021), as well as regulation of several physiological mechanisms and signalling pathways (Mottis *et al.*, 2019). This review combines new insights on mitochondrial function and addresses important and timely questions that remain unresolved. How do environmental stressors impact mitochondrial physiology? Which animal species, and which mitochondrial parameters, must be studied depending on the scientific questions asked? What is a good design to mimic environmental change and to test its effect on mitochondrial function? Does mitochondrial function correlate with whole-animal parameters, such as metabolic rate? Can we find a way to collect mitochondrial data properly in the field? And finally, how do we harmonise sample collection of threatened animals with conservation efforts? In other words, can we take advantage of mitochondrial physiology to understand/test the adaptive capacity of organisms, and can we develop a sustainable way to do so without threatening the organism we aim to preserve? Here, we address some of these questions and provide a toolbox for understanding how best to study mitochondrial function in organisms facing a changing world.

Climate change is a major threat currently faced by living organisms on our planet, disrupting the balance between energy availability and demand. Together with a generalised global warming and increased extreme temperature fluctuations, climatic models also predict variation in global precipitation patterns, as well as ocean acidification, deoxygenation, and salinification (Rogelj *et al.*, 2012; Masson-Delmotte, 2018; Bates and Johnson, 2020). All these events pose great challenges to organism physiology and performance, forcing changes in animal distribution, variation in trophic networks, but also population collapse and extinction (Pörtner and Farrell, 2008; Somero, 2010). The fundamental role played by aerobic metabolism in sustaining eukaryotic life, and the strong dependence of this process on both temperature and oxygen availability, make energy production and specifically mitochondrial physiology, a likely crucial determinant of animals’ ability to thrive in a changing environment. A better understanding of the extent by which the inability to sustain adequate mitochondrial energy production underpins failure of higher-level processes (e.g. cardiovascular failure) is crucial to both ecology and conservation studies.

Mitochondrial function is known to be affected by environmental changes, whether acute or long-term changes (Sokolova, 2018). Acute and extreme environmental variations, such as thermal variation (Thoral *et al.*, 2021; Thoral *et al.*, 2022b) or a fall in oxygen availability (Scott *et al.*, 2018; Dawson and Scott, 2022; Cerra *et al.*, 2023), can lead to mitochondrial changes linked to stress responses in order to cope with the shifting environment. On the contrary, environmental changes extending over long periods can lead to acclimation and adaptation of mitochondrial metabolism (Pichaud *et al.*, 2017; Camus *et al.*, 2017b; Bettinazzi *et al.*, 2024). Therefore, changes in mitochondrial function differ depending on the duration and intensity of the environmental variation as well as on the thermal strategy of their host (endotherms versus ectotherms). This suggests that, in some species, long-term changes, such as global warming, could sometimes be adequately managed at the mitochondrial (and individual) level (Steffen *et al.*, 2023). Indeed, as climate change also involves increased environmental fluctuations and extreme events such as heat waves, these rapid and unpredictable changes could be more deleterious for some animals (Masson-Delmotte, 2018). Thus, rapid and acute changes in temperature, oxygen availability, salinity, food availability and other parameters, as well as a combination of several variables at the same time can impact the physiology of the animals, particularly by affecting their mitochondrial function (Jørgensen *et al.*, 2021; Menail *et al.*, 2022; Menail *et al.*, 2023; Steffen *et al.*, 2023). Alongside other physiological parameters, mitochondrial function could therefore be one of the first to be modified in response to extreme variations in environmental parameters. These mitochondrial changes could then help determine if individuals are able to acclimatise to these new environmental conditions, which may or may not change in a predictable way, or lead to significant physiological stress, including respiratory dysregulation, oxidative stress and cellular senescence (Stier *et al.*, 2021).

Among the abiotic factors, temperature plays a central role in determining worldwide species distribution (Somero, 2005; Schulte, 2015). The thermal limits of mitochondrial performance might dictate whole-animal thermal tolerance and species distribution; for instance, heat sensitivity appears especially linked to OXPHOS failure, notably in the heart (Iftikar and Hickey, 2013; Christen *et al.*, 2020). Moreover, evidence of loss of mitochondrial performance and ATP production at temperatures close to the upper thermal limit is persistent in literature, especially for ectothermic species, whose metabolism is strictly linked with the external environment (Chung and Schulte, 2020). Evidence that mitochondrial function might determine whole-organism thermal susceptibility and biogeographical distribution ranges from intertidal copepods (Healy and Burton, 2023), insects (Jørgensen *et al.*, 2021; Lubawy *et al.*, 2022; Menail *et al.*, 2022), fish (Iftikar and Hickey, 2013; Iftikar *et al.*, 2014; Christen *et al.*, 2018), bivalves (Hraoui *et al.*, 2020; Hraoui *et al.*, 2021) and crabs (Iftikar *et al.*, 2010).

However, climate change is not solely constrained to the rise of temperatures and their fluctuation. Other phenomena such as the acidification and increased salinity of water (Melzner *et al.*, 2013; Cunillera-Montcusí *et al.*, 2022), or the impact of changes in precipitation frequency and intensity (McCluney *et al.*, 2012) could also affect mitochondrial function; it is thus essential to consider the multiple factors that underlie the impact of climate change on mitochondrial phenotype. Additionally, environmental extremes could exacerbate the main phenotypic impact of intergenomic incompatibility in naturally hybridising populations (Rank *et al.*, 2020; Bettinazzi *et al.*, 2024). It is therefore crucial to quantify interpopulation divergence at the level of interacting mitochondrial and nuclear genes (and therefore account for potential intergenomic incompatibilities) when testing local adaptation and population dynamics in a context of mutating environment (Ellison and Burton, 2008; Healy and Burton, 2020).

To study the effects of environmental variations on mitochondrial function, it is, first of all, essential to choose the most appropriate mitochondrial parameters to match the study species and questions, keeping in mind that measuring several parameters is always better to optimally assess mitochondrial function. For example, in an environment where oxygen and substrates are not limiting, the maximum oxidative capacity of mitochondria, their abundance and volume could be examined (Dawson *et al.*, 2022). The first can be measured through electron transfer system (ETS) complexes enzymatic activities (see table 1), while the second and third can be approximated through mitochondrial DNA copy number (Lubawy *et al.*, 2022) or the activity of enzymes such as citrate synthase (CS) (Larsen *et al.*, 2012; Milbergue *et al.*, 2022). However, if these parameters are limiting, then assessing mitochondrial efficiency to produce energy (Sappal *et al.*, 2015; Thoral *et al.*, 2021) may be more appropriate. Because temperature variation can affect several mitochondrial parameters, each with their own specific changing trajectories (Chung and Schulte, 2020; Dawson and Scott, 2022), it appears that multiple mitochondrial traits in parallel would ideally be considered to understand environmental effects on individuals (Metcalfe *et al.*, 2023). Combining measurements carried out at other biological scales to measure the relationship between mitochondrial metabolism and other functions such as immunity, thermal tolerance (Nord *et al.*, 2021), swimming performance (Thoral *et al.*, 2022a; Thoral *et al.*, 2024a), metabolic rate (Thoral *et al.*, 2024b), growth (Salin *et al.*, 2019; Dawson *et al.*, 2022), physical performance or activity (Bettinazzi *et al.*, 2024) and longevity (Camus *et al.*, 2023) of individuals and/or populations will provide a more complete picture of the relationship between environmental perturbations and organismal performance.

**Table 1:** Measures of mitochondrial function applied to a conservation framework and a (non-exhaustive) set of representative studies.

Mitochondrial parameter	Definition	Relevance	Techniques	Examples of studies
Mitochondrial oxidative phosphorylation (OXPHOS)				
Pathway-specific OXPHOS	Respiration in a specific electron transfer pathway (complex I, II or alternative electron pathways), in the OXPHOS (phosphorylating) state	Assess loss of efficiency, breakdown or preferential utilisation of specific pathway(s)	Respirometry (eg. Oroboros O2k, Clark-type electrodes, Seahorse XF), Probes (MitoXpress)	Stier *et al*., 2019, Gnaiger and MitoEAGLE Task Group, 2020 (for theoretical underpinnings), Wagner *et al*., 2019, Cossin-Sevrin *et al*., 2022, Rhodes *et al*., 2024
OXPHOS coupling efficiency	Coupling of electron transfer to phosphorylation of ADP to ATP. Calculated as: *jP-L* = (*P − L*)/*P* = 1 *− L/P* where *L* is LEAK, *P* is OXPHOS respiration	An index of mitochondrial efficiency to produce energy	Respirometry (eg. Oroboros O2k, Clark-type electrodes, Seahorse XF)	Gnaiger and MitoEAGLE Task Group, 2020, Dawson *et al*., 2022, Thoral *et al*., 2024a, McDiarmid *et al*., 2024
ADP/O	ADP consumed per oxygen consumed	An index of oxidative phosphorylation efficiency	Respirometry (eg. Oroboros O2k, Clark-type electrodes, Seahorse XF)	Kraffe *et al*., 2007, Sussarellu *et al*., 2013
ATP/O	ATP produced per oxygen consumed	Efficiency of ATP production per rate of respiration	High-resolution fluorespirometry (Oroboros O2k); kit-based assays (for eg. ATP hydrolysis and synthesis)	Salin *et al*., 2016b, Thoral *et al*., 2021, Roussel *et al*., 2018
Flux control ratios (FCRs)	Ratio of oxygen flux in a given respiratory state, normalised for a common reference state	Shows coupling and substrate control independently of mitochondrial content	Respirometry (eg. Oroboros O2k, Clark-type electrodes, Seahorse XF)	Bettinazzi *et al.*, 2019, Gnaiger and MitoEAGLE Task Group, 2020
Substrates contribution	Relative contribution of different energy metabolism pathways to mitochondrial respiration	Assess changes in the preferential utilisation of specific electron pathway(s)	Respirometry (eg. Oroboros O2k, Clark-type electrodes, Seahorse XF)	Jørgensen *et al.*, 2021, Rodríguez *et al.*, 2021
**Inner membrane characteristics**				
Membrane potential	Maintenance of mitochondrial membrane potential	Assessing the maintenance of the electron-proton circuit.	High-resolution fluorespirometry (e.g. Oroboros O2k); fluorescence microscopy and flow cytometry with cationic dyes (JC-1, Rhodamine 123, TMRM)	Lebenzon *et al.*, 2022, Harford *et al.*, 2023, Pouliot-Drouin *et al.*, 2024
Mitochondrial membrane composition	Lipid composition of the mitochondrial membrane (phospholipid classes, fatty acid abundance)	Assess the susceptibility of the mitochondrial membrane to oxidative damage, or temperature related changes (homeoviscous adaptations)	Lipidomics, GC-FID	Hazel, 1995, Munro and Blier, 2012

(Continued)

**Table 1:** Continued

Mitochondrial parameter	Definition	Relevance	Techniques	Examples of studies
**Mitochondrial oxidative balance**				
Mitochondrial reactive oxygen species (ROS) flux	ROS efflux (balance of production and consumption pathways) in a particular respiratory state	Dysregulation of these signalling molecules can lead to harmful defects	High-resolution fluorespirometry (eg. Oroboros O2k or other respirometers); spectrophotometric assays; flow cytometry; fluorescence microscopy	Loughland and Seebacher, 2020, Rodríguez *et al.*, 2021, Kienzle *et al.*, 2023, do Amaral *et al.*, 2021
Activity of enzymes linked with antioxidant capacity	Maximum activity of enzymes in isolation (e.g. SOD; CAT; GPx)	Shows maximal capacity of each enzyme	Spectrophotometric assays	Orr and Sohal, 1992, Doucet-Beaupré *et al.*, 2010, Pichaud *et al.*, 2010, Christen *et al.*, 2020, Loughland and Seebacher, 2020
Oxidative damage	Biological markers of oxidative damage (directly related or not to mitochondrial metabolism) to the membrane lipids, and the DNA	Assess the effects of oxidative damage	Kit-based assays: TBARS (for malondialdehyde species), 8-oxo-dG (for DNA)	Pelletier *et al.*, 2023a
**Activity of energy metabolism enzymes**				
OXPHOS complexes activity	Maximum enzymatic activity of specific ETS complexes in isolation (e.g. CI; CII; CI + CIII; CIV; ATPase))	Shows maximal capacity of each complex in isolation, outside of the ‘intact’ mitochondrial environment (potential pitfalls associated)	Spectrophotometric assays	Spinazzi *et al.*, 2012, Hunter-Manseau *et al.*, 2019, Rodríguez *et al.*, 2019, Bettinazzi *et al.*, 2021
Activity of enzymes linked with energy metabolism upstream of the ETS (linked with glycolysis, fermentation, fatty acid metabolism, intermediate metabolism, Krebs cycle)	Maximum activity of enzymes in isolation (e.g. PK; PFK; LDH; CPT; 3-hydroxyacyl-CoA dehydrogenase; PDH; PK; CS; MDH etc)	Shows the maximal capacity of each enzyme	Spectrophotometric assays	Pelletier *et al.*, 1994, Thibault *et al.*, 1997, Hunter-Manseau *et al.*, 2019, Bettinazzi *et al.*, 2021
Citrate synthase (CS) activity	Activity of a key enzyme of Krebs cycle, catalysing the conversion of acetyl-CoA and oxaloacetate to citrate	Proxies for the number of mitochondria, may show up- or down- regulation of mitochondrial content in the sample of interest	Kit or laboratory-made enzymatic assays	Bergmeyer, 2012, Larsen *et al.*, 2012

(Continued)

**Table 1:** Continued

Mitochondrial parameter	Definition	Relevance	Techniques	Examples of studies
**Mitochondrial protein content**				
Uncoupling proteins (UCPs)	Transporter proteins of the inner mitochondrial membrane that dissipate the proton gradient and reduce ATP production	Dissipation of the proton gradient as heat, as a protective mechanism against high ROS levels. Related to energy expenditure and efficiency of exercise and/or metabolism and thermal adaptation	mRNA expression; Western blotting	Hilse *et al.*, 2016, Bryant *et al.*, 2018
Mitochondrial protein content	Amount of mitochondrial proteins in purified mitochondrial isolates	Proxies for the number of mitochondria, may show up- or downregulation of mitochondrial content in the sample of interest	Kit or laboratory-made enzymatic assays	Pichaud *et al.*, 2010
**Structural or quantitative aspects of mitochondria**				
Supercomplex assemblies	Supramolecular organization of the electron transfer system	Structural assessment of the ETS organization, potential role in ROS, electron flux	Blue-native PAGE, proteomics	Bundgaard *et al.*, 2020
Cristae morphology	Mitochondrial cristae surface area and shape	Assess structural abnormalities and functional decline	Cryo-ET and EM	Perkins *et al.*, 2012, Brandt *et al.*, 2017
Mitochondrial DNA (mtDNA) copy number	Amount of mtDNA (relative to nuclear gene copies) can indicate increased mitochondrial abundance (or balance between mitogenesis and mitophagy)	Proxies for the number of mitochondria, may show up- or downregulation of mitochondrial content in the sample of interest	DNA extraction and qPCR	Ballard *et al.*, 2007, Meyer *et al.*, 2013, Noguera and Velando, 2019, Cossin-Sevrin *et al.*, 2022, McDiarmid *et al.*, 2024

See the main text for more examples of relevant articles.

## Mitochondrial function as a guide to population-level changes and conservation

Conservation science is continuously looking for ways to determine if a particular species or population is at risk due to changes in various biotic and abiotic factors. Assessing mitochondrial function could be a good approach to predict the effects of changes in habitat quality on individual health and potentially extend these predictions up to population and species fitness. Indeed, individuals with high quality mitochondria that can maintain efficient cellular energy production should be selected for (Fangue *et al.*, 2009; Iftikar *et al.*, 2014; Salin *et al.*, 2016b; Dawson *et al.*, 2022). However, how we measure mitochondrial quality remains context and species dependent. For example, having a greater number of mitochondria or a greater overall capacity to produce ATP would be beneficial to process large amounts of food when resources are abundant; however, when resources are low, sustaining a high level of food consumption may prove impossible (Salin *et al.*, 2016a; Salin *et al.*, 2019; Závorka *et al.*, 2021). The trend towards augmented mitochondrial efficiency rather than abundance appears to hold true for endotherms experiencing higher environmental temperatures (Fangue *et al.*, 2009; Dawson *et al.*, 2022), which increases energy requirements to sustain basic metabolic needs (Boyles *et al.*, 2011). Yet, endothermic animals inhabiting cold and challenging environments often increase capacity and reduce efficiency, possibly to produce heat (Nord *et al.*, 2021). These counterintuitive changes in mitochondrial phenotype in endothermic animals facing harsh environmental conditions are often accompanied by unique physiological specialisations in oxygen uptake, transport and usage (Scott *et al.*, 2010; Mahalingam *et al.*, 2017). Therefore, it is important to understand and identify what constitutes a ‘well suited’ mitochondrial phenotype, depending mainly on the environmental conditions experienced, but also on the species and tissue studied, and that could be defined by several parameters.

Increasing evidence points to mitochondrial function as a tool to guide conservation efforts, with studies in threatened species highlighting important roles for these organelles. For example, thermal stress in imperilled fish affects gene expression and alternative splicing related to mitochondrial processes (Thorstensen *et al.*, 2022). Unfavourable breeding conditions affect oxidative stress management and overall physiological condition, triggering compensatory behavioural adjustments in species of northern gannets (Pelletier *et al.*, 2023a; Pelletier *et al.*, 2023b). In contrast, other species such as king penguins can adjust mitochondrial function to cope with stress (Stier *et al.*, 2019). Mitochondrial ‘health’ can thus provide useful information on the feasibility of various conservation interventions targeting species and populations. Among these interventions, translocating populations can be a useful tool in conservation (George *et al.*, 2009), as well as genetic rescue through adaptive introgression (Hamilton and Miller, 2016). Introgressing foreign mitochondria with a lower mutational load or better adapted to a specific environment can potentially improve population performance and lead to adaptation (Hill, 2019). Nonetheless, evidence suggests that translocation can have catastrophic consequences on populations, as introducing incompatible mitochondrial variants might affect fitness (Ellison and Burton, 2006; Smith *et al.*, 2010; Innocenti *et al.*, 2011; Bettinazzi *et al.*, 2024) and potentially lead to extinction due to genetic incompatibility (Gemmell and Allendorf, 2001; Hughes *et al.*, 2003). Proper mitochondrial function necessarily relies on intergenomic coadaptation (Burton, 2022). For instance, interactions between mitochondrial and nuclear genes affect metabolic rate and organismal performance in response to temperature in both seed and leaf beetle species (Arnqvist *et al.*, 2010; Rank *et al.*, 2020), affecting their potential distribution and thermal adaptation. Successful conservation plans involving mitochondrial introgression should account for mitonuclear compatibility, and for that, a priori genetic screening and mitochondrial profiling of both parental and hybrid populations could be useful to test the potential of ‘mitonuclear outbreeding depression’ when planning genetic rescue of small, inbred populations. In line with this idea, the concepts of mitonuclear ecology and conservation mitonuclear replacement (CmNR) have recently emerged (Hill, 2015; Hill *et al.*, 2019; Iverson, 2024).

As illustrated by the famous Krogh’s principle (‘for a large number of problems there will be some animal of choice, or a few such animals, on which it can be most conveniently studied’) (Krogh, 1929), a natural system that is ideal to answer the question(s) of interest should be adopted when studying conservation physiology through the lens of mitochondrial physiology. Model species represent a powerful starting tool. For example, a versatile species like *Drosophila* which are easy to breed and maintain, and for which gold-standard genetic tools are widely employed (such as balancer chromosomes which prevent recombination, and various mutant lines readily available from stock centres) can enable researchers to select and study specific genetic variation of interest more precisely than in non-model species. Furthermore, model species are usually fast reproducing, making them a good tool to explore how environmental changes (including diet, which is easily modifiable) can impact the selection of mitochondrial phenotypes at various generational timescales (Camus *et al.*, 2017a; Camus and Inwongwan, 2023; Bettinazzi *et al.*, 2024). Fundamental principles about mitochondrial function can and have been discovered using laboratory animal models and can then be applied to other species in more natural settings, to help understand which selection pressures may act on populations in the wild (Rauhamäki *et al.*, 2014; Mesquita *et al.*, 2021; McDiarmid *et al.*, 2024).

Popular laboratory models that differ from the more traditional rodents and insect species include goldfish (Thoral *et al.*, 2022a; Thoral *et al.*, 2024a), zebrafish (Cadiz *et al.*, 2019; Thoral *et al.*, 2022b), zebra finches (Salmón *et al.*, 2023) or Japanese quails (Stier *et al.*, 2022). Species that are relatively easy to keep in laboratory conditions allows for unmatched sensitivity and control over the characterisation of the mechanisms underpinning mitochondrial function (like the *Drosophila* outlined above) which can then be harnessed to explore possible genetic/phenotypic selection pressures in wild systems. Indeed, recent years have seen a marked increase in the number of studies carried out on nontraditional species, such as wild animals captured in their natural environment and sometimes kept in captivity, including: brown trout (Dawson *et al.*, 2022); triplefin fish (Harford *et al.*, 2023); several species of birds (Barbe *et al.*, 2023a; Barbe *et al.*, 2023b); honey bees (Menail *et al.*, 2023); lampreys (Belyaeva *et al.*, 2014) and bivalves (Steffen *et al.*, 2023). The expanding diversity of study organisms and successes in determining their mitochondrial function would suggest that it should be possible to extend these measurements to most desired species to assess their energy metabolism. This would also allow us to discover common issues faced and strategies employed by species inhabiting similar habitats, or organisms facing similar pressures due to environmental change. In addition, the use of these wild species provides a wider genetic background than that obtained with species that have been kept in captivity for dozens or even hundreds of generations, which can affect certain physiological parameters such as their ability to acclimate (Morgan *et al.*, 2019).

## Resources and tools to study mitochondrial function in the laboratory and in the field

Researchers in the field of conservation physiology interested in characterising mitochondrial function in their species of interest might first be wondering which parameter(s) to target. To this end, we list some of the most informative parameters about mitochondrial function in Table 1. These are common and popular measurements, but researchers should keep in mind that finding clear distinction between ‘functional’ or ‘dysfunctional’ mitochondria might not be the definite objective. Indeed, there is a recent push to move mitochondrial science beyond function and dysfunction and better adapt the terminology to reflect that mitochondria are multifaceted, multifunctional, species and tissue-specific, dynamic organelles (see Monzel *et al.*, 2023 for a review and perspective).

The recognition of the pivotal role mitochondrial metabolism plays across an array of physiological processes (Garlid, 2001) along with the recent advancements in user-friendly technologies to study respiration (Gnaiger, 2011) and other mitochondrial parameters such as membrane potential (Pendergrass *et al.*, 2004) has resulted in substantial methodological improvements to measure mitochondrial function in the past decades. Multiple methods to assess mitochondrial parameters in different settings are available to researchers depending on their equipment, resources, time, and whether working in a fully equipped laboratory or in the field (see Palmeira and Moreno, 2018, for more details). Measurement of mitochondrial oxygen consumption rates provide a robust and detailed analysis of mitochondrial function, as the activity of specific enzymes, isolated mitochondria or permeabilized cells require different metabolic pathways for oxidation but all terminate in oxygen consumption (Makrecka-Kuka *et al.*, 2015; Vandenberg *et al.*, 2021). The two most commonly used instruments to measure mitochondrial respirometry use either chamber-based platinum electrodes (Seebacher and James, 2008; Rissoli *et al.*, 2017) or microplate-based fluorescence readings (Brand and Nicholls, 2011; Divakaruni *et al.*, 2014). These techniques are accessible for researchers with appropriate training obtainable either via one of the instruments’ manufacturers, or through collaboration with researchers in the ever-growing mitochondrial physiology field. The major advantage of both of these methods is the ability to produce real-time data that is not possible when using more traditional endpoint metabolic assays.

A popular instrument to measure rates of oxygen consumption using platinum electrodes in 0.5 ml or 2 ml chambers is the high-resolution respirometer Oxygraph-2 k (O2k, Oroboros Instruments, Innsbruck, Austria) that is available with an optional fluorescence module (O2k-Fluo). Over the last few years, dozens of different protocols have been set up and adjusted to measure various mitochondrial parameters on these O2k oxygraphs (Blier and Lemieux, 2001; Stier *et al.*, 2017b; Teulier *et al.*, 2019; Bettinazzi *et al.*, 2019b; Dawson *et al.*, 2020b; Thoral *et al.*, 2021; Harford *et al.*, 2023; Nord *et al.*, 2023; Rodríguez *et al.*, 2023; Steffen *et al.*, 2023). This instrument’s advantages are the assessment of several parameters of mitochondrial function simultaneously from a single sample, i.e. oxygen consumption and either ATP production (Magnesium Green; Thoral *et al.*, 2021), ROS production (Amplex UltraRed/Ampliflu Red; Steffen *et al.*, 2023), calcium uptake (Calcium Green; Cheng *et al.*, 2023) or membrane potential (TMRM, Harford *et al.*, 2023). These devices also allow acute monitoring of several parameters, including temperature and oxygen concentration. The different chamber sizes available also allow different biological samples to be studied, from isolated mitochondria (Christen *et al.*, 2018; Barbe *et al.*, 2023b) to whole individuals or cells (Patil *et al.*, 2013), as well as permeabilized, shredded or homogenised tissue samples (Dawson *et al.*, 2020b; Thoral *et al.*, 2022b). An equally popular alternative to the O2k is the Seahorse XF, which offers greater use for high-throughput analysis and is often used in the biomedical field (Divakaruni *et al.*, 2014). This instrument can measure far more samples simultaneously when compared to the O2k using 8- to 96-well plates and offers measures of mitochondrial respiration, glycolysis and ATP production in cells and isolated mitochondria. However, it does not measure the fluorescence-based process outlined above simultaneously and conditions inside the sample well such as oxygen saturation cannot be as readily modified as in the O2k chambers. Thus, these different techniques offer a wide range of possibilities for studying mitochondrial function, depending on the species studied, the biological samples used and the researchers’ budget.

As mentioned previously, numerous methods for preparing mitochondria for analysis exist, ranging from intact cells (Nord *et al.*, 2021) with mitochondrial respiratory uncouplers and inhibitors that can permeate through the plasma membrane, to detergent- or mechanically permeabilized tissues and cells (Dawson *et al.*, 2020b), and even isolated organelles to assess fine kinetic function or different subpopulations of mitochondria (Scott *et al.*, 2018; Rodríguez *et al.*, 2020; Dawson and Scott, 2022). Each method offers advantages and disadvantages that must be carefully evaluated. Indeed, permeabilized tissues can be useful to circumvent the collection of high quantities of tissue and minimise the use of other instruments (such as centrifuges) that are needed for mitochondrial isolation (Kuznetsov *et al*., 2008). Isolated mitochondria, however, are taken out of their cellular context and are thus free of many of the confounding biochemical pathways and processes that may interfere or compete with mitochondrial function, such as ATPases or NADH-consuming enzymes. Working with tissue samples and blood cells can be done with minimal manipulation, and can in some cases provide a non-terminal method of sampling individuals, ideal for longitudinal studies (Stier *et al.*, 2017a; Stier *et al.*, 2019; Stier *et al.*, 2022; Nord *et al.*, 2023; Thoral *et al.*, 2024a; Thoral *et al.*, 2024b).

Although the typical mitochondrial respirometry-based approaches outlined above rely on fresh samples, access to these is not always possible and the cryopreservation of tissues for later analysis of mitochondrial function can sometimes be a more desirable solution. A key problem is the effect of freeze thawing of mitochondria on their membranes and subsequent functionality of intact mitochondria. Recently developed approaches in wild animals (Bettinazzi *et al.*, 2019a) provide a solution to this. Assessing mitochondrial function, with the measure of mitochondrial respiration for example, using minimal amounts of mitochondria is now possible in frozen mitochondria, tissue and cells (2–30 μg of isolates or homogenates, or 30 000 cells per well; Acin-Perez *et al.*, 2020). This analysis on previously frozen samples relies on carefully optimised conditions including the addition of NADH as a substrate (rather than pyruvate, glutamate and malate) to assess complex I activity, along with a need for pre-incubation with succinate for complex II respiration. It also comes with a major limitation, as the freeze thawing of samples causes an uncoupling from oxidative phosphorylation since ATP synthase can no longer exert control over respiration (Acin-Perez *et al.*, 2020).

Another means of measuring mitochondrial function on previously frozen samples is the measurement of enzymatic activities (Spinazzi *et al.*, 2012) which are commercially available in assay kits and straightforward to replicate. The assessment of enzyme capacities from frozen tissues is particularly useful when working in remote locations or time- and resource-constrained situations (Dawson *et al.*, 2020a; Schell *et al.*, 2023). Enzyme assays have a potential drawback in that they are assessed under *in vitro* conditions that deviate from physiological settings (pH, osmolarity, substrate concentrations, etc.) and they do not allow the evaluation of respiratory coupling. Nonetheless, they offer vital quantitative data regarding catalytic and flux capacities of the mitochondrial respiratory complexes where more complex respirometry experiments are not possible. The levels of the different ETS complexes can also be compared through western blotting techniques and can reveal key changes in mitochondrial organisation (Jové *et al.*, 2014). Finally, accurate measurement of mitochondrial content remains a topic of debate. Indeed, CS activity or mitochondrial copy number are a commonly used proxies of mitochondrial content across tissues and species (Larsen *et al.*, 2012); however, a recent method using mitochondrial-targeted nLC-MS/MS (nano-liquid chromatography/mass spectrometry) might provide more accurate measurements of this crucial parameter (McLaughlin *et al.*, 2020).

## Perspectives, pitfalls and perorates

The diverse range of stressors explored herein (temperature, oxygen availability, salinity, food availability) create an imbalance between energy requirements and energy production, highlighting the potential importance of studying differences in mitochondrial energy production as a potential tool to help guide conservation efforts. However, what is unclear from a conservation standpoint is how different organisms can cope with changes in the environment both temporally and in intensity. The main aim of conservation practices should therefore be to determine how quickly a population or species can react to acute environmental shifts, if they can acclimate in case these changes are permanent, and what the consequences are of possible phenotypic selection acting on these populations.

As such, it is critical to identify what constitutes a ‘high quality’ mitochondrial phenotype for each species, population and environment in order to properly guide conservation efforts. The works presented herein highlight a potential issue with lab-based studies in that mitochondrial function is almost always analysed under highly controlled conditions that can be quite distinct from the physiological conditions in which mitochondria naturally exist.

In the case of studying mitochondrial thermal sensitivity (specifically when trying to link the upper thermal limit of organisms with their mitochondrial thermal performance), one concern lies in the choice of substrates given to the mitochondrial preparation. Indeed, at a temperature close to their thermal limit, Jørgensen and colleagues showed that some *Drosophila* species exhibit a breakdown in Complex I-linked respiration (Jørgensen *et al.*, 2021). However, maximal coupled respiration was maintained through the oxidation of alternative substrates such as glycerophosphate, even when exposed to temperatures above their thermal limit. Thus, the careful dissection of the different steps of the ETS is paramount, as this potential ‘rescue’ of respiration by alternative complexes at higher temperatures might not be accompanied by sufficiently efficient ATP production (as not all mitochondrial complexes pump protons and hence differ in their contribution to ATP production). Respiration protocols used to guide conservation efforts must therefore be as informative as possible on the relative contributions of each ETS complex, and measure ATP production rates and/or ROS efflux to properly assess mitochondrial quality.

Moreover, at the individual level, laboratory conditions are also generally far from the natural conditions in which individuals live, due to their stability and/or the precise control of different environmental parameters. Future projects should therefore focus on new approaches to get as close as possible to the physiological and environmental conditions experienced naturally by individuals (Drake *et al.*, 2017), while bearing in mind that the conditions for *in vitro* measurements of mitochondrial function are likely distinct from natural conditions due to the technical limitations of the instruments used and should be confirmed with wild studies (Dawson *et al.*, 2016; Nord *et al.*, 2021). Therefore, it is important to initially consider what the stable conditions representative of the organism under study are, and to compare the biotic or abiotic factors under question to these ‘standard’ conditions (e.g. stickleback and temperature studies in Cominassi *et al.*, 2022 and Dawson *et al.*, 2022).

Recent progress has been made by several groups looking at linking mitochondrial function to traditionally studied parameters. One of these important traits is metabolic rate, a key parameter measured in studies concerned with conservation. It seems that the relationship between mitochondrial function and whole-animal metabolic rate depends on many factors such as the organ, tissue or cellular compartment of interest; the type of preparation used for respirometry; the status of the individual (for e.g. stress) and the parameter (respiration state) or mitochondrial pathway under scrutiny (Malkoc *et al.*, 2021; Cominassi *et al.*, 2022; Casagrande *et al.*, 2023). Even behavioural traits such as territorial ‘performance’, linked to standard metabolic rate (Metcalfe *et al.*, 1995) have been correlated to maximal OXPHOS capacity and mitochondrial density (Larsen *et al.*, 2012). Other fitness traits such as reproductive output can also be linked to mitochondrial function. For example, male reproductive success can be impaired by temperature in *Drosophila* (Van Heerwaarden and Sgrò, 2021); and studies on temperature and other stressors have shown that reproductive tissue quality (number of eggs, sperm motility, etc.) can vary with mitochondrial function, often measured in the same tissue (Bettinazzi *et al.*, 2019b; Bettinazzi *et al.*, 2020; Rank *et al.*, 2020; Bettinazzi *et al.*, 2023; Camus *et al.*, 2023). Investigating mitochondria should therefore go beyond the traditional dichotomy between ‘function’ and ‘dysfunction’ when being used in a conservation setting: mitochondrial phenotypes, behaviour, features and activities (Monzel *et al.*, 2023) can and should be investigated from the perspective of overall animal performance (see Heine and Hood, 2020 for a review and a hypothesis). Moreover, and closely linked to mitochondrial physiology (due to mitochondria’s central role as a metabolic hub), there is a need for other approaches and markers such as oxidative status (Beaulieu and Costantini, 2014) and metabolomics analysis (Lawson *et al.*, 2022) to be used in conservation strategies, with some successful examples recently published in birds and in mussels (Putnam *et al.*, 2023; Waller *et al.*, 2023; Pelletier *et al.*, 2023a; Pelletier *et al.*, 2023b).

Finally, the example of wild species captured and then brought back to the laboratory shows the current limits that scientists face in studying energy metabolism. The different measurement methods presented above require stable conditions which are mainly found in the laboratory. However, it seems essential to find solutions so that these measurements take place directly in the field, in order to allow the non-terminal measurements of individuals in the wild (Dawson *et al.*, 2016), by taking small samples of tissues such as muscle biopsies (Quéméneur *et al.*, 2022; Thoral *et al.*, 2024a) or blood samples (Stier *et al.*, 2017b; Nord *et al.*, 2021), while also releasing individuals back into their natural environment immediately after collection. Although some scientists have already started experimenting with portable field laboratories such as the ‘MitoMobile’ (Parry *et al.*, 2021), it now seems essential to continue to devise new methods and techniques that can be easily brought into the field to study mitochondrial function with the aim of guiding and improving conservation efforts, without terminally sampling from the very populations we aim to protect.

## Data Availability

No data were generated or analysed during this work.
